# Enhancing betacyanin stability: Comparison of dragon fruit (*Hylocereus polyrhizus*) pulp and peel powders through encapsulation technology during storage

**DOI:** 10.1002/fsn3.3992

**Published:** 2024-02-20

**Authors:** Bambang Nurhadi, Muhammad Abdillah Hasan Qonit, Syariful Mubarok, Rudy Adi Saputra

**Affiliations:** ^1^ Department of Food Industrial Technology, Faculty of Agro‐Industrial Technology Universitas Padjadjaran Sumedang West Java Indonesia; ^2^ Department of Agribusiness Darul Ma'arif University Indramayu West Java Indonesia; ^3^ Department of Agronomy, Faculty of Agriculture Universitas Padjadjaran Sumedang West Java Indonesia

**Keywords:** betacyanin, dragon fruit peels, dragon fruit pulps, encapsulation, stability

## Abstract

Betacyanin can be found in the peel or pulp of dragon fruit. As a natural pigment, betacyanin is unstable, so it requires encapsulation technology to maintain its quality. The stability of encapsulated betacyanin from dragon fruit peel compared to dragon fruit pulp has yet to be discovered. This study aims to compare the stability of encapsulated betacyanin (with maltodextrin and gum Arabic) from dragon fruit peel and pulp dried with vacuum drying. Dragon fruit peel extraction utilized a 50% aqueous ethanol solvent, while pulp juice extraction was performed. The ratio of dragon fruit extract to coating materials was set at 1:3 (solid/solid). Research shows that dragon fruit juice powder had higher stability and phytochemical concentrations than the ethanol extract of dragon fruit peel powder during 30 days of storage. Despite similar color stability (similar range value of ΔE), the color from dragon fruit juice powder more closely resembled the natural fruit, albeit with weaker antioxidant activity than the peel powder. Betacyanin concentration in juice powder was notably higher (82.56–156.82 μg/g) than in the ethanol extract of dragon fruit peel powder (52.51–75.12 μg/g). A combination of maltodextrin and Arabic gum (1:1) as coating materials demonstrated the highest concentrations of total phenolic and total betacyanin (81.15–95.87 mg/g and 121.91–156.82 μg/g, respectively) during the storage period. These findings contribute to our comprehension of betacyanin stability and functionality, facilitating precise applications in industrial processing environments based on their source attributes.

## Graphical abstract

The linear response of peel‐derived betacyanin contrasts with the relatively fluctuating trend observed in puree‐derived betacyanin, attributed to the intricate compound composition of puree‐based powder. Coating with maltodextrin in manufacturing yields drier powders, enhancing shelf stability, particularly in maltodextrin‐enriched peel‐derived powders with elevated phenolic content. The synergistic effect of maltodextrin and gum arabic further enhances phytochemical levels in pulp‐derived powders, advancing our understanding of betacyanin's stability and potential application and promising increased efficacy of dragon fruit's essential betacyanin components.

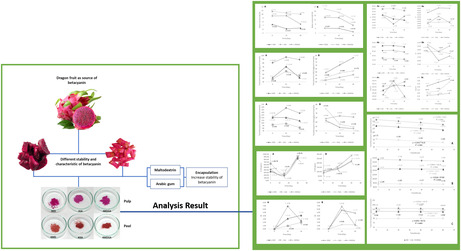

## 1 INTRODUCTION

Betacyanin is a water‐soluble pigment containing nitrogen utilized in the industry as a natural dye due to its appealing red‐purple hue (Sadowska‐Bartosz & Bartosz, 2021). In pharmaceuticals, betacyanin has anti‐inflammatory, anti‐cancer, anti‐lipidemic, detoxifying, and anti‐microbial properties (Giridhar, 2017). Each commodity as a source of betacyanin has a different composition and characteristic of a complex system with a different focus on antioxidant properties (Bastos & Schliemann, 2022). Red dragon fruit is one of the natural commodities that can be utilized as a source of betacyanin. Dragon fruit, or *Hylocereus polyrhizus*, is a horticulture product of the cactus family with a distinctive fruit shape and numerous properties, especially antioxidants (Lira et al., 2020). The advantage of dragon fruit's betacyanin content is that it has a less earthy flavor and aroma than beets due to its lower geosmin and pyrazine content (Thi et al., 2019). Besides that, dragon fruit peel is often unused and ends up as waste. According to Jalgaonkar et al. (2020), dragon fruit waste, such as dragon fruit peel, still has many properties, such as being a source of betacyanin. However, the characteristics of betacyanin between dragon fruit pulp and dragon fruit peel are unknown.

The low level of stability possessed by betacyanin in dragon fruit is an impediment. As a pigment derived from nature, betacyanin is susceptible to pH levels, temperature, air exposure, and light, limiting its use (Sawicki et al., 2019). Due to the oxidative stress caused by excessively high temperatures, the betacyanin content could degrade when exposed to excessively high temperatures, resulting in a lower shelf life for betacyanin (Otálora et al., 2020). The leading cause of the low stability of betacyanin is due to the reversibility and degradability of betalain molecules, which undergo breakdown to betalamic acid and cyclo‐DOPA (5,6‐dihydroxyindoline‐2‐carboxylic acid) after oxidation and undergo Schiff base condensation in betanin regeneration at low‐temperature conditions (Chew et al., 2019). Furthermore, the breakdown of betacyanin forms dopachrome and cyclo‐DOPA 5‐O‐D‐glucoside, which are radicals, while betanidin forms betanidin quinone due to oxidation with peroxyl radicals (Sadowska‐Bartosz & Bartosz, 2021). The unstable nature of betacyanin makes its industrial application challenging. By encapsulating betacyanin, it is possible to increase its stability.

Encapsulation is a technology that provides physical protection to the core material (solid, liquid, and gas) by covering it with a coating material to avoid direct contact between the core material and environmental conditions (Abd El Kader & Abu Hashish, 2020). The use of encapsulation technology can increase the stability of the core material from oxidizing agents such as environmental conditions (light, temperature, humidity, and high oxygen concentrations), reduce the rate of evaporation of volatile compounds, and control the rate of release of the core material (Huang et al., 2023). This technology has been widely used to increase the stability of core materials, which are sensitive either because they are volatile or prone to oxidation (Zabot et al., 2022). Previously, many applications of encapsulation technology had been carried out on betacyanin from dragon fruit peels with various coating materials, such as in a study by Rahayuningsih et al. (2021) and Fathordoobady et al. (2021). However, there is no comparison of stability properties between dragon fruit juice and peel with various encapsulating materials. In addition, this coating technology can also reduce a material's hygroscopicity (Coy‐barrera, 2020). Applying encapsulation technology to betacyanin is strongly advised since producing a protective layer or coating can prevent the degradation of pigment compounds caused by external influences (Castro‐Enríquez et al., 2020). The coating material, which functions as a protective layer, is a crucial aspect of encapsulation that must be considered.

The coating material must cover the active ingredients, be non‐reactive with the active ingredients, retain the active ingredients during processing or storage, and release solvents during encapsulation (Fabela‐Morón et al., 2022). Maltodextrin and arabic gum are popular coating ingredients used in encapsulation. The addition of maltodextrin and Arabic gum to a substance does not enhance its sweetness but can enhance the transition glass temperature (*T*_*g*_) of the material, allowing the product to remain stable during storage (Stępień et al., 2022). The high *T*_*g*_ value of the encapsulation material can increase product stability, reduce product stickiness, and increase the yield and efficiency of the encapsulation process (Agatha et al., 2021). Increasing pigment stability through encapsulation has been demonstrated in various studies. In the research of Vargas‐Campos et al. (2018), encapsulation was able to increase the color stability of dragon fruit powder for more than one month. Meanwhile, research by Otálora et al. (2023) shows that encapsulation of the betaxanthin pigment in gummy candy can increase the stability of color and improve texture, such as gumminess and chewiness. This study aims to compare the stability of betacyanin from the peel and flesh of dragon fruit, which has been encapsulated and dried using maltodextrin and Arabic gum as coating materials/fillers.

## 2 MATERIALS AND METHODS

### 2.1 Materials

Fresh red dragon fruit with a perfect red peel is obtained from a local market in Bandung, Indonesia. The Maltodextrin DE 10–12 supplier was Qinhuangdao Lihua Starch Co., Ltd. (China). Kimia Market in Bandung, Indonesia, was purchased for food‐grade arabic gum and 96% technical ethanol. Ethanol absolute p.a. (pro analysis), acetone p.a., Folin–Ciocalteu reagent, sodium acetate trihydrate p.a., glacial acetic acid, potassium chloride p.a., citric acid monohydrate p.a., standard gallic acid, and methanol absolute p.a. were purchased from Merck, USA. The ABTS assay kit and sodium carbonate were acquired from Sigma‐Aldrich in the United States.

### 2.2 Raw material preparation

Fresh red dragon fruit whose original weight has been measured. After the pulp and fruit peel are separated, their weights are determined. Begin preparing dragon fruit peel extract by slicing the fruit peel to 1 × 5 cm and dehydrating it at 60°C for 8 h in a food dehydrator. The dragon fruit peel powder was produced by grinding and sieving the dried products to a 60‐mesh size. The next step was extraction using the maceration method with 50% ethanol solvent at a ratio of 1:10 between the powdered dragon fruit peel and the solvent, which took 24 h in dark conditions. The maceration results were then filtered and evaporated using a BÜCHI rotary vacuum evaporator R‐300 at 50°C and 80 revolutions per minute (rpm) in a rotary vacuum evaporator to generate a liquid extract of dragon fruit peel until the moisture content remaining was 10% (at constant weight conditions). The pulp resulting from separation from the peel is then separated between the juice and the seeds using a slow juicer.

### 2.3 Dragon fruit peel extract and juice encapsulation

The resulting dragon fruit peel extract and juice were then combined with three different coating materials, namely maltodextrin (JMD for juice and KMD for peel extract), arabic gum (JGA for juice and KGA for peel extract), and a mixture of maltodextrin and arabic gum (1:1) (JMDGA for juice and KMDGA for peel extract) with a raw material‐to‐coating material ratio of 1:3. The resulting mixture of raw materials and coating ingredients was then dried for 10 h at 50°C and 0.08 MPa in a B‐One VOV‐50 Vacuum Drying Oven (China). The drying products were then crushed for 60 s to generate encapsulated powders of dragon fruit juice and dragon fruit peel extract.

### 2.4 Sample conditioning storage treatment

The powder encapsulated from the juice and ethanol extract of dragon fruit peel powder is wrapped in a zip‐lock bag made of aluminum foil. The packing results were stored in a desiccator at 22–24°C with humidity regulated to 10% using silica gel blue. Storage is performed for one month, and sampling is performed every 15 days.

### 2.5 Moisture stability analysis

The water content analysis followed the procedure based on AOAC (AOAC, 2005). The study was conducted at 0 days, 15 days, and 30 days of storage to determine the stability of the moisture content during storage. The analysis was conducted by weighing a 5 g sample in a cup of constant weight. The sample was baked in an oven at 105°C for 30 min, and the process was repeated until a steady weight was achieved. The results of measuring the water content are the water content on a dry basis (Equation 1):

(1) $$\text{Moisture content}\;\left(\mathrm{dry}\;\text{basis}\right)=\frac{b-\left(c-a\right)}{\left(c-a\right)}\times 100\%$$

*a* = dry cup mass (g). *b* = initial sample mass (g). *c* = dry sample mass + dry cup (g).

### 2.6 Stability analysis of total phenolic content

The total phenolic test was carried out using the Folin–Ciocalteu method with gallic acid as a standard. The activity carried out in this test was making dragon fruit samples by dissolving dragon fruit powder in ethanol solvent with a concentration of 4000 ppm or 4 mg/mL, which was stirred using a magnetic stirrer and filtered using filter paper. The sample solution was put into a 25‐mL volumetric flask measuring 1 and 0.5 mL of Folin Ciocalteu 1:1 reagent (Folin and water) and 2.5 mL of 20% sodium carbonate reagent. The solution in the volumetric flask was stirred gently, adjusted with distilled water, and then incubated in a dark place for 40 min. The incubation results were read on a spectrophotometer at a wavelength of 725 nm. Analysis was carried out at three storage times, namely 0 days, 15 days, and 30 days, to determine the stability of the total phenolic content during storage.

### 2.7 Betacyanin total stability analysis

Total betacyanin stability was evaluated over three different storage durations (0, 15, and 30 days). Betacyanin was analyzed using a 50% ethanol solvent (absolute p.a. + distilled water (1:1)) to ensure its quality. 100 mg of powdered dragon fruit was weighed using an analytical balance before being dissolved in 25 mL of 50% ethanol. The solution was filtered through Whatman No. 41 filter paper and placed in a dark container to prevent light‐induced oxidation. The solution was tested using a UV/Vis spectrophotometer (UV‐1700 Shimadzu, Japan) at 536 nm for total betacyanin and 600 nm for the browning correction factor (Sitompul et al., 2019). To calculate total betacyanin (Equation 2) (Priatni & Pradita, 2015):

(2) $$\text{Total betacyanin}\;\left(\left(\mathrm{\mu g}\right)/g\right)=\frac{A\left(\mathrm{MW}\right)V\left(\mathrm{DF}\right)1000}{\mathrm{ELW}}$$

A, Absorbance (538 nm – 600 nm); DF, Dilution factor; L, Cuvette length (cm); V, Extract volume (mL); W, Sample weight (g); E, Mean molar absorptivity = 6.5 × 104 L/mol cm in H_2_O (for bethanin). MW, Molecular weight = 550 g/mol.

### 2.8 Measurement of total betacyanin stability against pH

Testing the stability of betacyanin to pH was carried out at pH 3, 4, and 5. Tests were done by making distilled water adjusted to pH 3, 4, and 5 using 0.2 M citric acid and 0.1 M disodium hydrogen phosphate. 4.5 mL of the prepared stock solution was sampled into a test tube, and 0.5 mL of the sample dissolved in 50% ethanol was added and vortexed until homogeneous. The mixing results were read on a spectrophotometer with 538 nm and 600 nm wavelengths. Readings are taken every 20 min for 1 h.

### 2.9 Color stability analysis

Based on the CIELAB standard, color analysis was performed using a Konica Minolta Head CR‐400 colorimeter. Measurements derived L*, a*, and b* values. Examiners were undertaken at 0, 15, and 30 days of storage to evaluate the color stability during storage. Calculation yields the degree value of Hue (H°) (Equation 3), while calculation yields Chroma (C*) (Equation 4):

(3) $$H^{0}=\frac{\tan^{-1}b^{*}}{a^{*}}$$

(4) $$C^{*}=\sqrt{a^{2}+b^{2}}$$

### 2.10 Antioxidant activity stability analysis

The stability of antioxidant activity was determined by doing an ABTS assay at three different times during storage, utilizing protocols based on the studies of Aboagye et al. (2021) and Mareček et al. (2017) with some modifications. For the production of ABTS solution and potassium persulfate, a 0.2 M acetate buffer containing sodium acetate trihydrate was employed as the solvent. The pH was adjusted with glacial acetic acid to 4.5. To create the ABTS^+^ reagent, combine 7 mM aqueous ABTS and 2.34 mM aqueous potassium persulfate in a 1:1 ratio. Before use, the ABTS^+^ reagent was incubated in the dark for 16 h. After incubation, the reagent was diluted with a prepared acetate buffer and measured with a spectrophotometer until the absorbance at 734 nm was optimally 0.7. The ABTS^+^ reagent must be used immediately since storage causes the absorbance value to drop. With a 50% ethanol solvent, the sample solution for fruit was made at five concentrations: 100, 50, 25, 12.5, and 6.25 ppm. As a result of dilution and homogenization, the analytical solution was generated by combining 1 mL of sample solution at each concentration with 2 mL of ABTS^+^ reagent. After homogenization, the analytical solution was incubated for 10 min in the dark, and the absorbance at 734 nm was measured. A combination of 2 mL of diluted ABTS^+^ reagent and 1 mL of acetate buffer served as the blank. Using the reference absorbance (A_0_) and sample absorbance (A_s_) to obtain the % inhibition value using the formula (Equation 5).

(5) $$\%\text{inhibition}=\frac{A_{0}-A_{s}}{A_{0}}\times 100$$

The IC_50_ value was determined utilizing a data set of % inhibition from various sample concentrations to generate a linear equation with intercept and slope values. IC_50_ is determined using the following formula (Equation 6).

(6) $${\mathrm{IC}}_{50}=\frac{\left(50-\text{intercept}\right)}{\text{slope}}$$

### 2.11 Statistical analysis

A comprehensive statistical analysis was undertaken to assess the stability of quality attributes in the ethanol extract of dragon fruit peel powder and dragon fruit juice powder. The consistency of these attributes within the samples was evaluated using the standard deviation. Subsequently, Pearson correlation analysis was conducted using the established SPSS 25.0 statistical software to unveil relationships between various quality parameters. These findings were visualized using a heatmap, effectively simplifying intricate data patterns and highlighting interconnections among the variables.

## 3 RESULT AND DISCUSSION

### 3.1 Stability of moisture content

**FIGURE 1:**
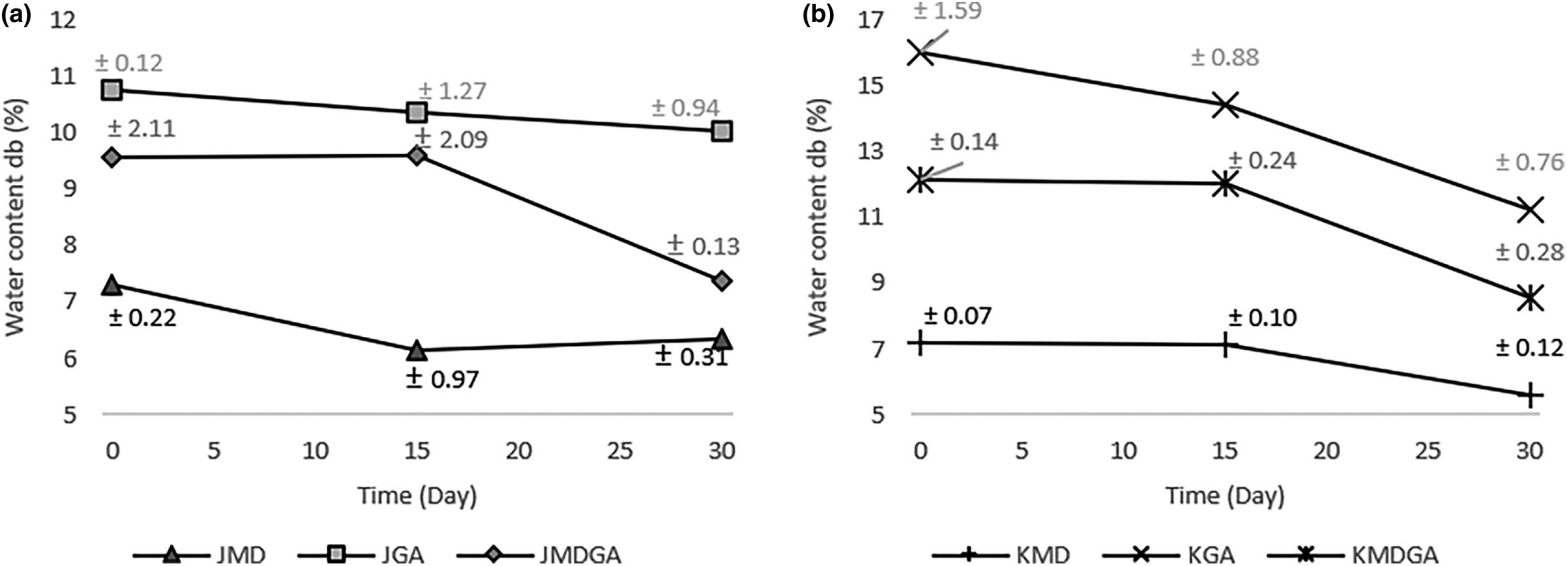
Moisture content (dry basis) in dragon fruit juice powder (a) and ethanol extract of dragon fruit peel powder (b) encapsulated at three storage times. JMD, Juice + Maltodextrin; JGA, Juice + Arabic gum; JMDGA, Juice + Maltodextrin + Arabic gum; KMD, ethanol extract of dragon fruit peel + Maltodextrin; KGA, ethanol extract of dragon fruit peel + Arabic gum; KMDGA, ethanol extract of dragon fruit peel + Maltodextrin + Arabic Gum.

The presence of moisture can lead to undesired effects like clumping due to liquid bridges forming between particles, as Chen et al. (2019) demonstrated. Notably, the ethanol extract of dragon fruit peel powder has a higher water content than powdered dragon fruit juice (Figure 1). Both the ethanol extract of dragon fruit peel powder and juice powders usually exhibit an initial water level of at least 10%. Nevertheless, Zambrano et al. (2019) indicate that the ideal moisture content for dry food and powdered products is <10%. This outcome could be attributed to the relatively low drying temperature (approximately 50°C at 0.08 MPa pressure), impeding complete water release. Over time, the water content of each juice powder and ethanol extract of dragon fruit peel powder decreased relative to the initial levels, reflecting the low humidity (10%) and temperature (22–24°C) conditions in the storage desiccator. This environment induces desorption of the material's water content (Pham & Le, 2018), aligning with the principle that materials’ relative humidity tends to equilibrate with their surroundings (Luampon & Charmongkolpradit, 2019). Among them, the treatment with the lowest change during storage was the gum arabic treatment, with a decrease of 6.87% in juice powder. Meanwhile, in the ethanol extract of dragon fruit peel powder, the treatment with the lowest change was the maltodextrin application treatment, with a change of 22.09%.

Regarding the maltodextrin coating, it exhibited the lowest water content among treatments. Elevated maltodextrin ratios led to the formation of amorphous structures and reduced water content, likely due to diminished hygroscopicity (Wang et al., 2021) and decreased hydrophilicity. Dextrins, with their fundamental 1,6‐glycosidic and 1,4‐glycosidic linkages, are known for their substantial water‐binding properties (Włodarczyk & Śliżewska, 2021). Incorporating maltodextrin into the fruit material resulted in minimal water loss during storage. Conversely, the arabic gum treatment exhibited the highest water content in juice powder and the nethanol extract of dragon fruit peel powder, declining over time. Augmenting arabic gum, a coating agent, on dragon fruit hindered moisture loss due to its high hygroscopic nature, leading to a moisture‐rich substance (Karangutkar & Ananthanarayan, 2020). This effect arises from arabic gum's strong hydrophilic groups, facilitating water absorption from the surroundings and inducing hydrolysis (Lima et al., 2019).

### 3.2 Stability of total phenolic content

**FIGURE 2:**
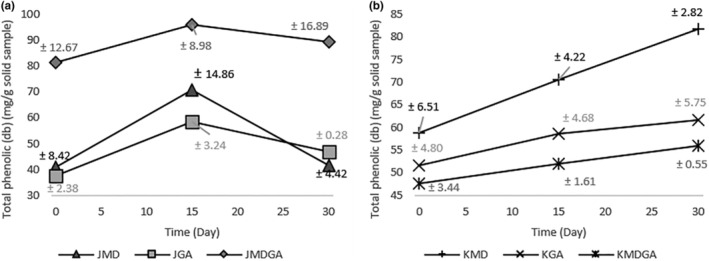
Total phenolic (dry basis) in dragon fruit juice powder (a) and ethanol extract of dragon fruit peel powder (b) encapsulated at three storage times. JMD, Juice + Maltodextrin; JGA, Juice + Arabic gum; JMDGA, Juice + Maltodextrin + Arabic gum; KMD, ethanol extract of dragon fruit peel + Maltodextrin; KGA, ethanol extract of dragon fruit peel + Arabic gum; KMDGA, ethanol extract of dragon fruit peel + Maltodextrin + Arabic Gum.

Figure 2 visually represents the outcomes of the total phenolic analysis. The information provided shows that the total phenolic content in the juice powder and ethanol extract of dragon fruit peel powder varies conspicuously. Specifically, the total phenolic content of juice powder initially increased over 15 days and subsequently decreased over the ensuing 15 days. Dragon fruit juice powder has a relatively higher total phenolic concentration, reaching 95.87 mg/g, compared to the ethanol extract of dragon fruit peel powder, which only has a total phenolic content of 81.71 mg/g. Based on its stability, the changes in juice powder are lower than the ethanol extract of dragon fruit peel powder. The percentage change in the total phenolic content in juice powder was the lowest at 1.85% when maltodextrin was added, while in the ethanol extract of dragon fruit peel powder, the lowest was 17.49% when the combination of coating ingredients was added. Conversely, the total phenolic constituents in the ethanol extract of dragon fruit peel powder exhibited an increment throughout a 30‐day storage period. Betacyanin in dragon fruit belongs to the phenolic group and changes during storage (Paśko et al., 2021). The breakdown of betacyanin in dragon fruit gives rise to additional phenolic compounds with labile attributes, including byproducts like betalamic acid and diverse cyclo‐DOPA compounds such as cyclo‐DOPA malonyl‐glucoside and 2‐decarboxy‐cyclo‐DOPA, as indicated by Slimen et al. (2017).

Heating triggers a Millard interaction between tyrosine protein and sugar in dragon fruit juice powder, inducing oxidative traits that diminish total phenolics, known for their antioxidant function (Iriondo‐DeHond et al., 2020; Troise, 2018). Meanwhile, the ethanol extract of dragon fruit peel powder is processed to reduce non‐betacyanin chemicals. Notably, incorporating arabic gum into the treatment resulted in a lesser decline than the non‐arabic gum treatment. According to Ćujić‐Nikolić et al. (2018), the anthocyanin content stabilized in the presence of arabic gum during heating and storage. In contrast, the combined use of maltodextrin and arabic gum exhibited slower changes than other approaches. Utilizing arabic gum as a supplementary matrix enhances material resilience and guards against oxidation, paralleling the phenolic content protection observed in propolis (Sukri et al., 2021). The gradual release mechanism of arabic gum contributes to a decrease in measurable phenolic content (Ćujić‐Nikolić et al., 2018; Lopes et al., 2019).

### 3.3 Total betacyanin stability

**FIGURE 3:**
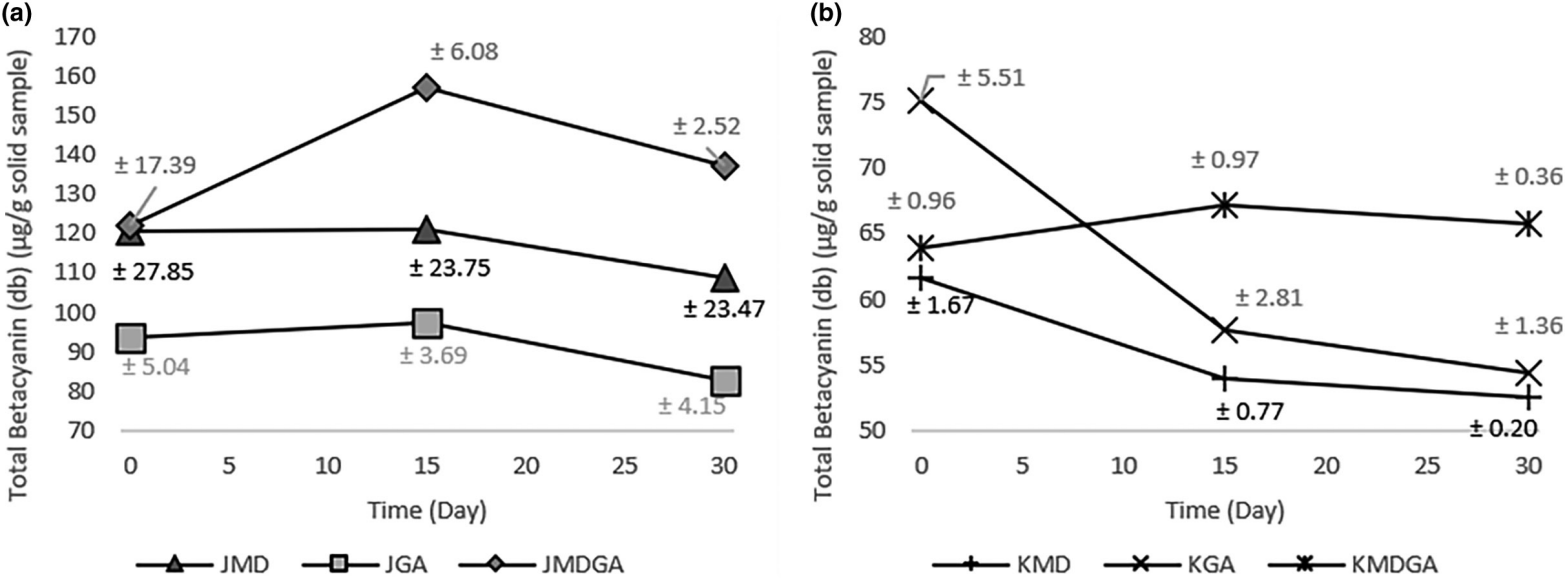
Total betacyanin (dry basis) in dragon fruit juice powder (a) and ethanol extract of dragon fruit peel powder (b) encapsulated at three storage times. JMD, Juice + Maltodextrin; JGA, Juice + Arabic gum; JMDGA, Juice + Maltodextrin + Arabic gum; KMD, ethanol extract of dragon fruit peel + Maltodextrin; KGA, ethanol extract of dragon fruit peel + Arabic gum; KMDGA, ethanol extract of dragon fruit peel + Maltodextrin + Arabic Gum.

This decline is evident in Figure 3, where the betacyanin concentration in encapsulated dragon fruit powder diminishes over time due to its inherent vulnerability. These data show that the total concentration of betacyanin in juice powder is superior to the ethanol extract of dragon fruit peel powder. The maximum concentration in juice powder reached 156.82 μg/g, while the ethanol extract of dragon fruit peel powder only reached 75.12 μg/g. Overall, juice powder has better total betacyanin stability with a change range of around 9.71% to 12.5%, while the ethanol extract of dragon fruit peel powder reaches 2.91% to 27.64%. Based on the coating material, the combination of ingredients triggered an increase in total betacyanin during storage, with an increase of 12.5% in juice powder and 2.91% in ethanol extract of dragon fruit peel powder. At the same time, other treatments experienced a decrease during storage. The maltodextrin and arabic gum blend initially increased on day 15 and then decreased by day 30, in contrast to the decreasing trend in other treatments. These findings indicate that a 1:1 mixture of maltodextrin and arabic gum maintains stable betacyanin levels during storage. Lima et al. (2019) observed that this combination augments the preservation of encapsulated natural colors, like anthocyanins, in *Euterpe edulis* fruit. Arabic gum as a secondary matrix enhances material resilience against oxidation (Sukri et al., 2021). The protective effect of this mixture of maltodextrin and gum arabic reduces the susceptibility of cyclo‐DOPA to changes, resulting in a consistent, stable total betacyanin concentration during the first 15 days for juice powder and ethanol extract of dragon fruit peel powder.

The decrease in total betacyanin value during storage is caused by various damage processes, such as aldimine bond cleavage, dehydrogenation, deglycosylation, decarboxylation, and isomerization (Karangutkar & Ananthanarayan, 2020). The shift from purplish red to brownish yellow signifies betacyanin's degradation (Leong et al., 2018). Dehydrogenation gradually alters betacyanin's hue from purplish red to yellow, forming compounds like betalamic acid and cyclo‐DOPA through sugar or phenolic transformations (Gengatharan et al., 2016; Sadowska‐Bartosz & Bartosz, 2021). Notably, cyclo‐DOPA 5‐O‐glucoside and betalamic acid are less stable than betacyanin (Grewal, 2020).

### 3.4 Antioxidant activity stability

**FIGURE 4:**
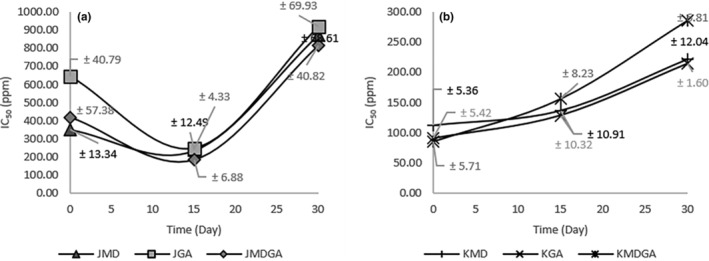
IC50 values of dragon fruit juice powder (a) and the ethanol extract of dragon fruit peel powder (b) encapsulated at three storage times. JMD, Juice + Maltodextrin; JGA, Juice + Arabic gum; JMDGA, Juice + Maltodextrin + Arabic gum; KMD, ethanol extract of dragon fruit peel + Maltodextrin; KGA, ethanol extract of dragon fruit peel + Arabic gum; KMDGA, ethanol extract of dragon fruit peel + Maltodextrin + Arabic Gum.

Betacyanin prominently features antioxidant attributes (Bastos & Schliemann, 2020). The strength of antioxidant activity is characterized using the IC_50_ value (Inhibition Concentration 50); a lower IC_50_ number signifies more potent antioxidant activity. Antioxidant activity is classified as essential if the IC_50_ value is ≤50 ppm, moderate if the IC_50_ = 101–150 ppm, weak if the IC_50_ = 151–200 ppm, and extremely weak if the IC_50_ > 200 ppm (Manurung et al., 2017). Based on these criteria, the gathered data (Figure 4) indicates moderate to weak antioxidant activity in encapsulated powders, juice powders, and ethanol extracts of dragon fruit peel powders.

Overall, the antioxidant activity of the ethanol extract of dragon fruit peel powder is relatively more potent than that of powdered juice samples. The ethanol extract of dragon fruit peel powder had a lower IC50 value of 85.89 ppm (parts per million) compared to juice powder, which had a value of 183 ppm. The lower the IC50 value, the stronger the antioxidant activity. However, based on its stability, juice powder experiences lower changes during storage, ranging from 42.59% to 149.67%, compared to the ethanol extract of dragon fruit peel powder, which experiences 98.84% to 233.76%. Antioxidant activity wanes with storage time, aligning with Nugrahani et al. (2020) finding of declining activity over extended periods. In Figure 4, the antioxidant activity of juice encapsulation powder surged in the initial 15 days and declined by day 30. Encapsulation of a powdered ethanol extract of dragon fruit peel decreased both antioxidant activity and storage duration. The upsurge in antioxidant activity within juice powder after 15 days stems from component decomposition into by‐products with enhanced antioxidant potency.

Elevated temperatures and processing times can diminish antioxidant potential. Herdyastuti et al. (2021) discovered that excessive drying temperatures elevated the IC_50_ value, signifying reduced antioxidant activity. Comparatively, the ethanol extract of dragon fruit peel powder exhibited higher antioxidant activity than the juice‐encapsulated powder, owing to the greater betacyanin concentration in ethanol extract of dragon fruit peel powder. Juice powder contains non‐antioxidant components like sugar. Dragon fruit's betacyanins degrade into molecules with antioxidant attributes, including betalamic acid, cyclo‐DOPA, and their derivatives (Grewal, 2020; Miguel, 2018). However, the oxidation products of betacyanin contain unstable betalamic acid and cyclo‐DOPA derivatives that quickly decarboxylate (Nakagawa et al., 2018). The decarboxylation of betalamic acid produces 6‐decarboxy‐betalamic acid (Kumorkiewicz et al., 2020; Martínez‐rodríguez et al., 2022). The decrease in dragon fruit juice powder's antioxidant activity after 30 days is attributed to the heat‐induced breakdown of betacyanin components into derivatives, thus lowering antioxidant activity in the ethanol extract of dragon fruit peel powder during storage (Sadowska‐Bartosz & Bartosz, 2021). Due to the unstable properties of a derivative of betacyanin, the antioxidant activity of dragon fruit peel powder decreases over time.

### 3.5 Color stability

**FIGURE 5:**
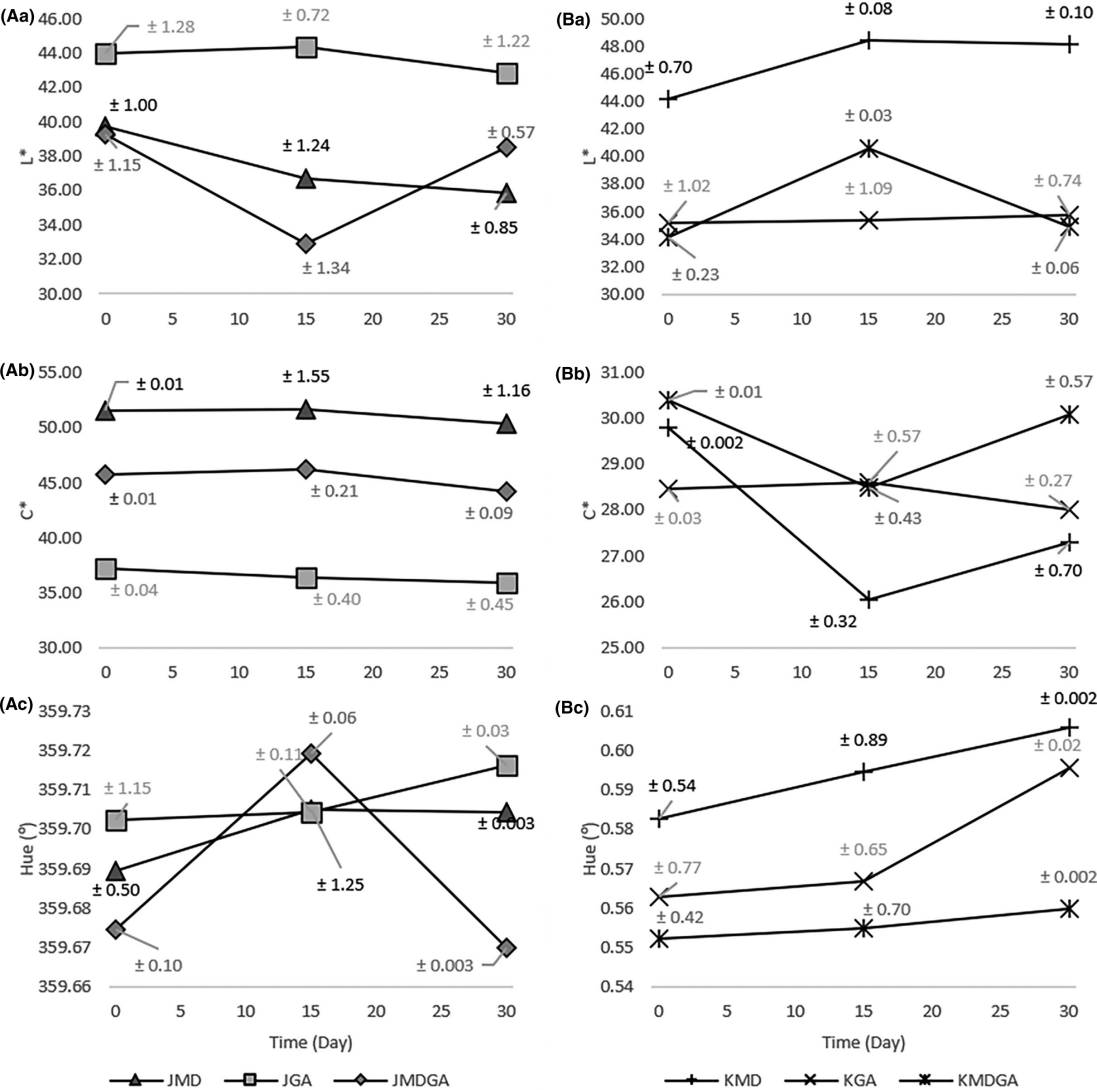
The color of dragon fruit juice powder in L* (Aa), C* (Ab), Hue (Ac) values and ethanol extract of dragon fruit peel powder in L* (Ba), C* (Bb), Hue (Bc) values encapsulation results at three storage times. JMD, Juice + Maltodextrin; JGA, Juice + Arabic gum; JMDGA, Juice + Maltodextrin + Arabic gum; KMD, ethanol extract of dragon fruit peel + Maltodextrin; KGA, ethanol extract of dragon fruit peel + Arabic gum; KMDGA, ethanol extract of dragon fruit peel + Maltodextrin + Arabic Gum.

The betacyanin content within dragon fruit undergoes color shifts as oxidation progresses (Kumorkiewicz et al., 2019). The color evaluation gauges the effectiveness of encapsulation technology in safeguarding natural pigments. Figure 5 illustrates the utilization of L*, C*, and ^0^H values to characterize an object's color.

The L* value indicates lightness, with lower values indicating darker shades and higher values denoting lighter ones. Notably, the L* value for juice powder and ethanol extract of dragon fruit peel powder follows a discernible pattern with storage duration. This inverse association aligns with the betacyanin content and the color's L* value. Elevated betacyanin content in dragon fruit powder correlates with reduced L* values. Moreover, color can be influenced by water content and relative humidity (RH). Jalgaonkar et al. (2020) demonstrated that increased RH and water content could induce color changes in red dragon fruit powder.

**FIGURE 6:**
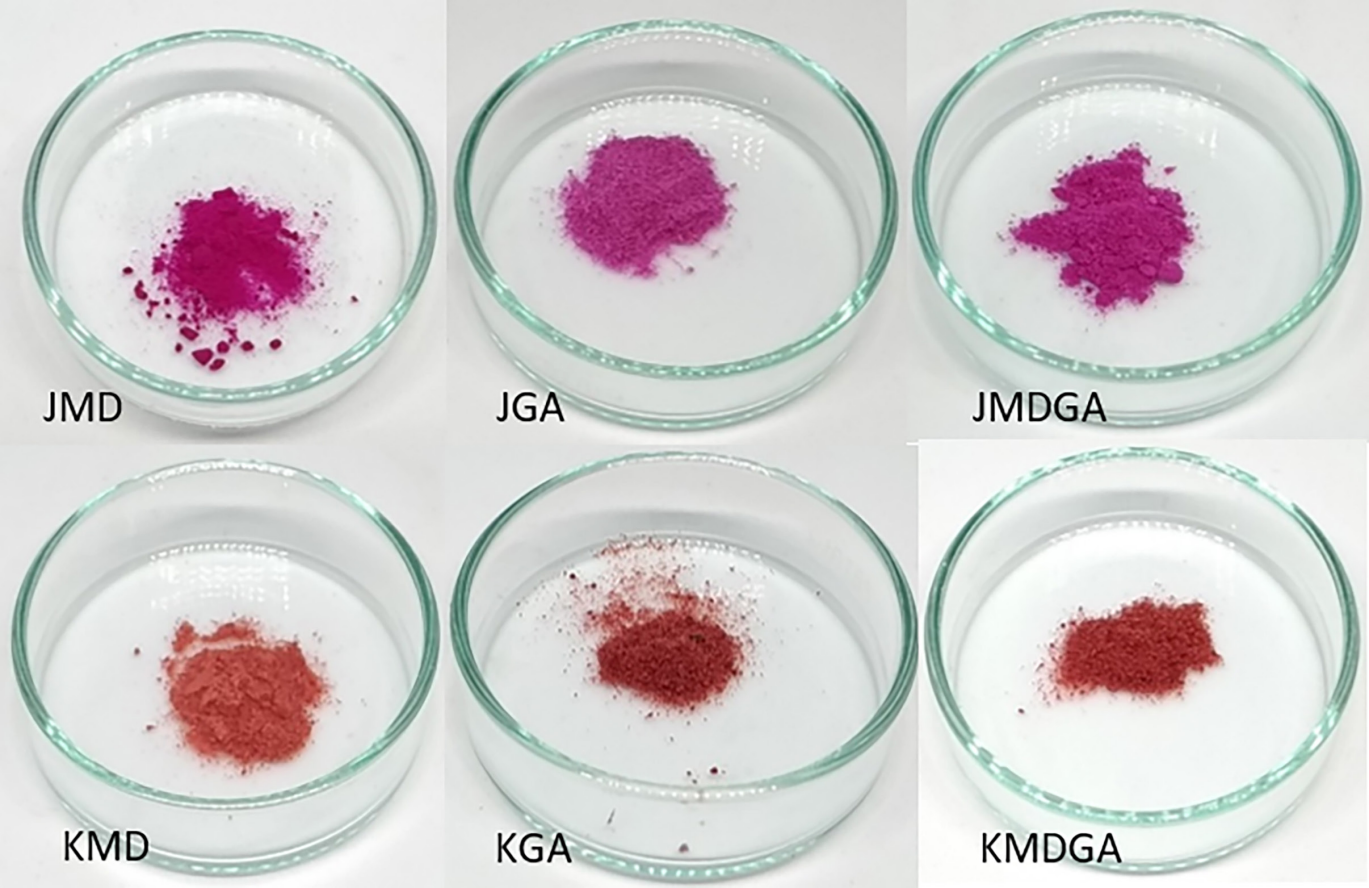
Dragon fruit juice powder and ethanol extract of dragon fruit peel powder in various coating materials. JMD, Juice + Maltodextrin; JGA, Juice + Arabic gum; JMDGA, Juice + Maltodextrin + Arabic gum; KMD, ethanol extract of dragon fruit peel + Maltodextrin; KGA, ethanol extract of dragon fruit peel + Arabic gum; KMDGA, ethanol extract of dragon fruit peel + Maltodextrin + Arabic Gum.

Comparatively, arabic gum treatment outperforms maltodextrin addition in terms of stability. Notably, dragon fruit juice powder treated with arabic gum exhibited the highest L* value compared to other treatments. This brownish‐white hue of arabic gum interacts with betacyanin's color, differing from maltodextrin (Figure 6). In contrast, the higher L* value in maltodextrin treatment for the ethanol extract of dragon fruit peel stemmed from the somewhat brownish‐yellow hue of the product, reflecting against the white color of maltodextrin. Maltodextrin's presence amplifies the L* value more significantly due to its white color, which diminishes the native dragon fruit hue as maltodextrin content increases. Despite the concealment of arabic gum's brownish hue, it becomes visible in the sample's color (Figure 6). With prolonged storage, the substance's color encapsulated in arabic gum fades, gradually revealing the color of the arabic gum matrix.

Meanwhile, the low L* value in the ethanol extract of dragon fruit peel powder treated with arabic gum results from a reaction between cyclo‐DOPA and arabic gum. Interactions between dragon fruit's betanidin and cyclo‐DOPA structures (which contain catechol) and arabic gum's anionic polysaccharides yield dark‐colored melanin (Huang et al., 2021; Le & Le, 2021). Discoloration can also result from changes in the material composition beyond the coating substance. Betacyanin can alter its hue by producing colorless cyclo‐DOPA 5‐O‐glucoside and bright yellow betalamic acid (Rodriguez‐amaya, 2018).

Chroma value (C*ab), which quantifies color intensity. Greater chroma values yield sharper colors, while lower values manifest as darker colors. For juice powder samples, no changes occurred across various treatments. However, the powdered ethanol extract of dragon fruit peel showed a reduction in chroma value in the initial 15 days, followed by an increase in the subsequent 15 days. In the latter period, the arabic gum treatment exhibited declining chroma values, resulting from betalamic acid yellowing due to interaction with arabic gum, yielding dark‐colored melanin (Huang et al., 2021; Le & Le, 2021).

Hue categorizes color's resultant appearance. In the sample of juice powder, the Hue value (^0^) across all treatments corresponded to purplish‐red. The arabic gum treatment showcased the best Hue value on both day 0 and day 30. Over 15 days, the Hue values for juice powder treated with maltodextrin and the maltodextrin + arabic gum blend increased. Subsequently, the arabic gum treatment maintained relatively consistent Hue values, while the mixed treatment experienced a substantial decline. The Hue value was highest for the ethanol extract of dragon fruit peel powder treated with maltodextrin. In all treatments, including maltodextrin, arabic gum, and their combinations, a rise in storage time was correlated with increased Hue values, particularly evident in arabic gum treatment from day 15 to day 30.

**FIGURE 7:**
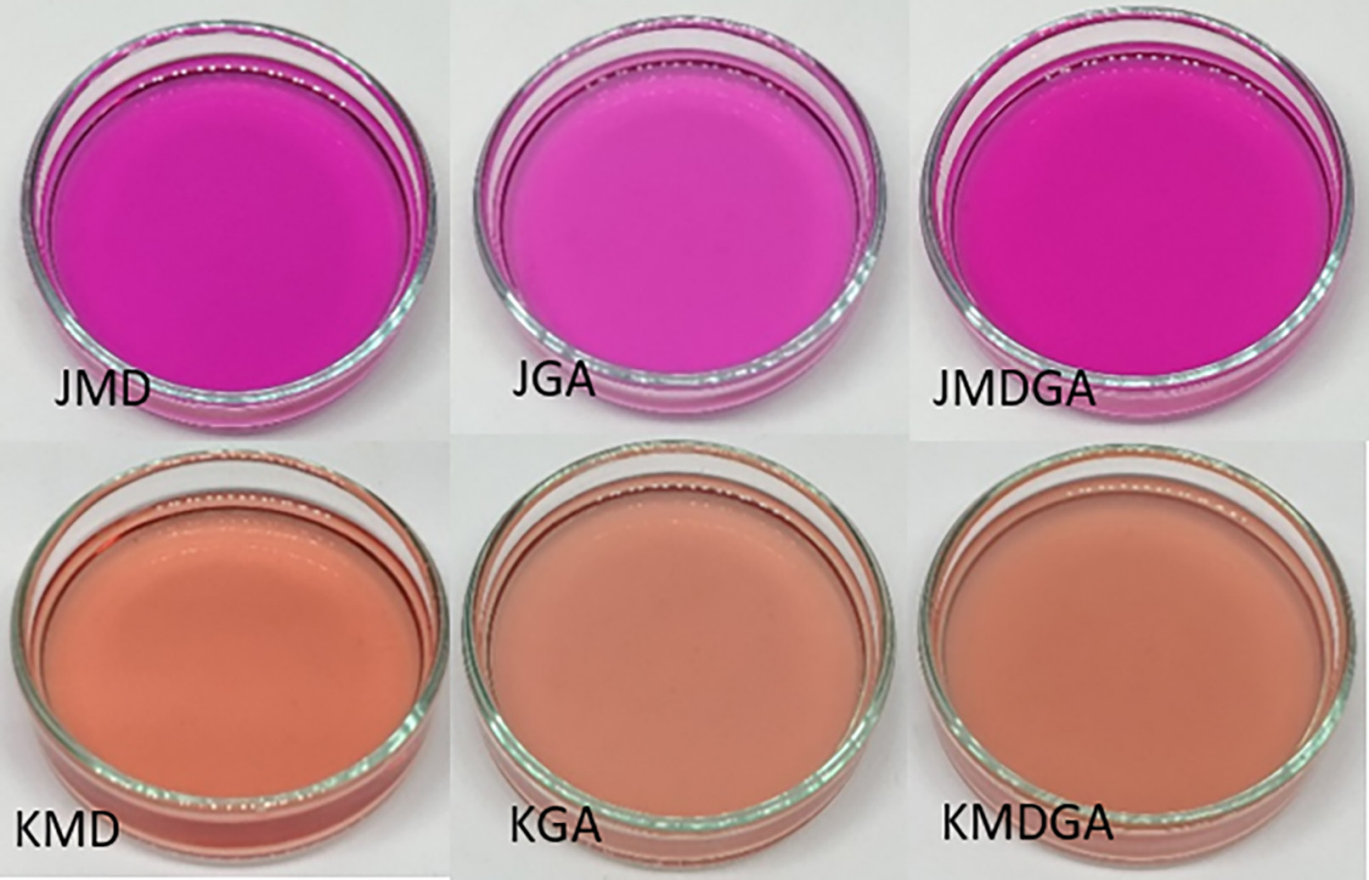
The color gradation of rehydrated juice powder and ethanol extract of dragon fruit peel powder is based on the coating material. JMD, Juice + Maltodextrin; JGA, Juice + Arabic gum; JMDGA, Juice + Maltodextrin + Arabic gum; KMD, ethanol extract of dragon fruit peel + Maltodextrin; KGA, ethanol extract of dragon fruit peel + Arabic gum; KMDGA, ethanol extract of dragon fruit peel + Maltodextrin + Arabic Gum.

Juice powder displayed a more pronounced purplish hue post‐rehydration than the ethanol extract of dragon fruit peel powder (Figure 7). Attractive colors significantly influence consumer perception (Luo et al., 2019; Rathee & Rajain, 2019). High‐contrast colors are generally preferred over pale or colorless alternatives (Paakki et al., 2019), making dragon fruit juice powder more viable for natural food coloring than the ethanol extract of dragon fruit peel powder.

**FIGURE 8:**
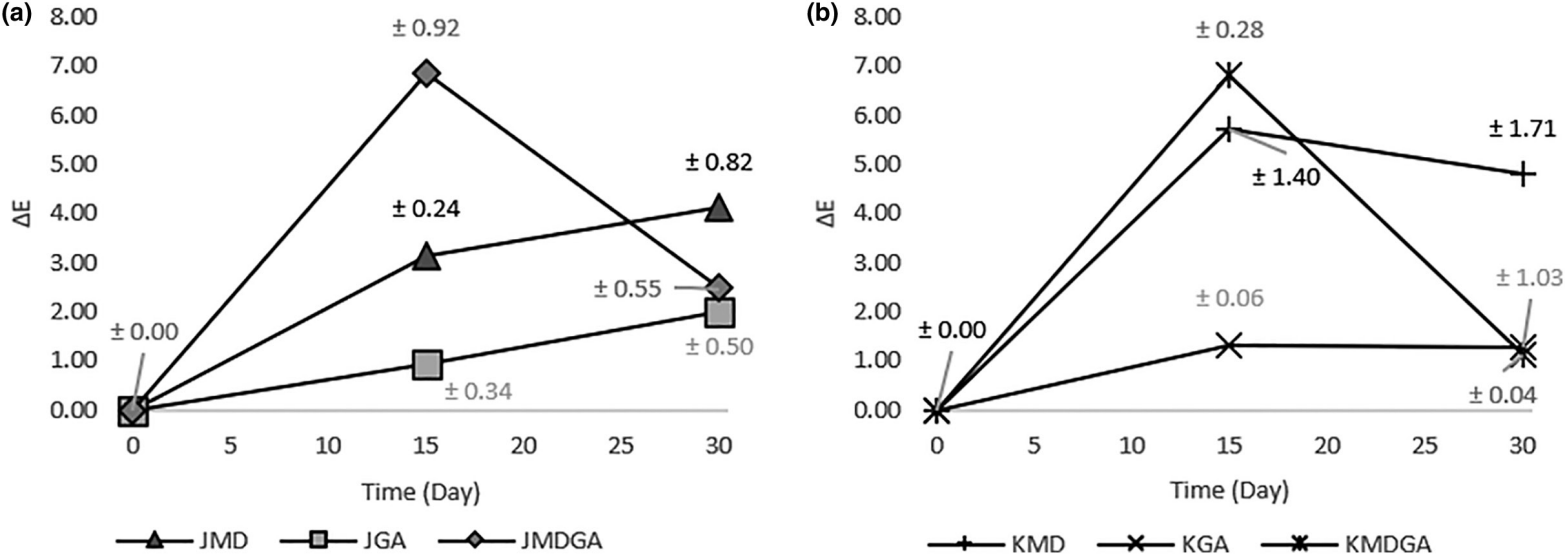
Δ*E* value of dragon fruit juice powder (a) and ethanol extract of dragon fruit peel powder (b) encapsulated at three storage times. JMD, Juice + Maltodextrin; JGA, Juice + Arabic gum; JMDGA, Juice + Maltodextrin + Arabic gum; KMD, ethanol extract of dragon fruit peel + Maltodextrin; KGA, ethanol extract of dragon fruit peel + Arabic gum; KMDGA, ethanol extract of dragon fruit peel + Maltodextrin + Arabic Gum.

Apart from L*, C*, and ^0^H, the Δ*E* value, combining a*, b*, and L* parameters, tracked color changes during storage. Δ*E* signifies color alteration, with lower values indicating minor shifts and higher values reflecting significant ones. The Δ*E* value scale's implications are presented in Table 1, with Figure 8 portraying Δ*E* value observations.

**TABLE 1:** The range of ΔE values in determining the degree of color difference from storage on day 0.

Δ*E* value	Description/definition
0–1	Differences that are usually not visible
>1–2	A very small difference, visible only to a trained eye
>2–3.5	Moderate differences, too, are obvious to the untrained eye
>3.5–5	Obvious difference
>5	Very clear difference

Over the initial 15 days, Δ*E* values and signaling color shifts in juice powder increased, notably in the maltodextrin + arabic gum blend. Subsequently, this shift diminished in the combined treatment, whereas other treatments continued to experience changes due to rising Δ*E* values. All treatments manifested color shifts linked to ΔE value increases in the ethanol extract of dragon fruit peel powder samples, particularly in maltodextrin and mixed treatments. In the ensuing 15 days, Δ*E* values dropped in maltodextrin and mixed treatments, with the mixed approach showing the slightest alteration. The arabic gum treatment exhibited an increased Δ*E* value, although the color impact during storage remained minor.

### 3.6 Pearson heatmap correlation between dragon fruit powder quality stability parameters

**FIGURE 9:**
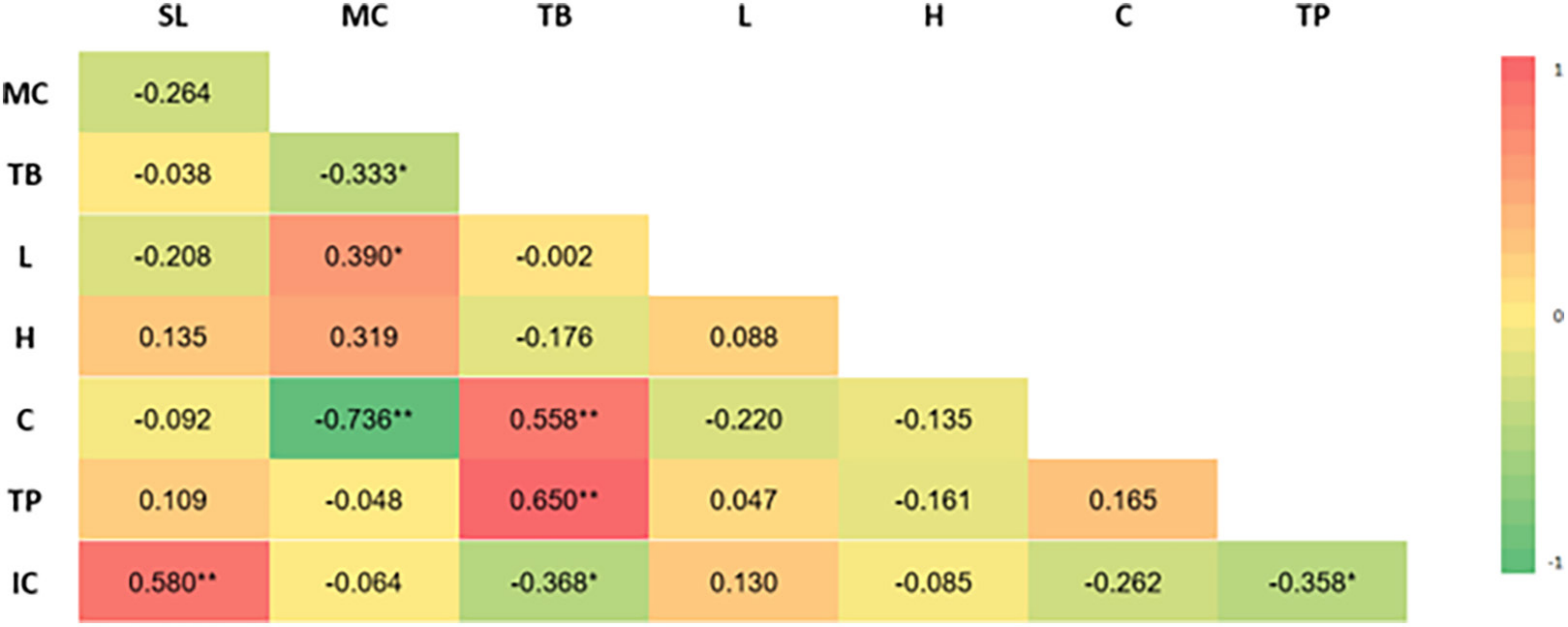
The Pearson correlation heatmap of the quality stability of dragon fruit juice powder. The quality stability parameters encompass Storage Time (ST), Moisture Content (MC), Total Betacyanin (TB), L* (L), Hue (H), C* (C), Total Phenolic (TP), and IC50 (IC). The color scale ranges from −1 (indicating a strong negative correlation, represented by green) to 1 (indicating a strong positive correlation, represented by red).

**FIGURE 10:**
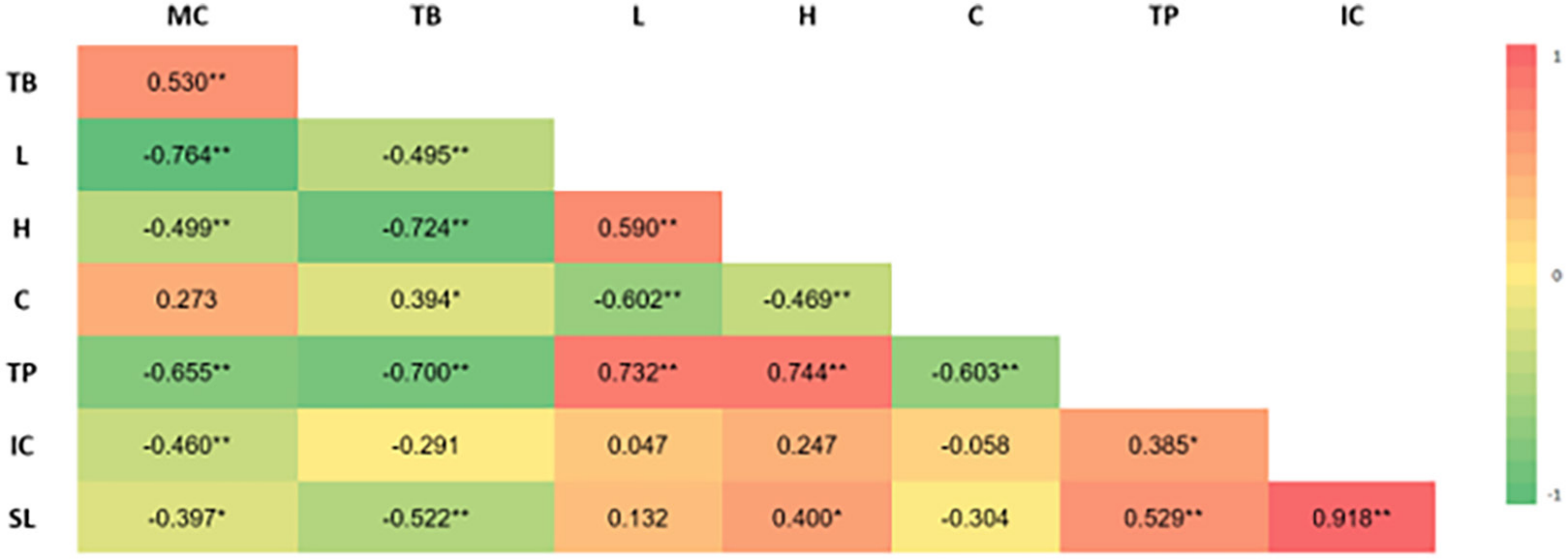
The Pearson correlation heatmap of the quality stability of ethanol extract of dragon fruit peel powder. The quality stability parameters encompass Storage Time (ST), Moisture Content (MC), Total for Review Only Betacyanin (TB), L* (L), Hue (H), C* (C), Total Phenolic (TP), and IC50 (IC). The color scale ranges from –1 (indicating a strong negative correlation, represented by green) to 1 (indicating a strong positive correlation, represented by red).

The Pearson correlation coefficient can be used to measure the relationship between variables. Results are considered to be significantly correlated if the *p*‐value is more than ±0.30 to ±0.49 and very significant if the *p*‐value is more than ±0.50 to ±1. Pearson correlation analysis examines storage time, water content stability, total phenolic stability, total betacyanin stability, antioxidant (IC_50_) stability, and color stability (L*, Hue, C*) in the ethanol extract of dragon fruit peel powder and juice powder. This analysis is presented as a heatmap, with Pearson's correlation coefficients for these stability parameters depicted in Figure 9 for dragon fruit juice powder and Figure 10 for the ethanol extract of dragon fruit peel powder.

For juice powder (Figure 9), significant correlations are observed: SL positively correlates with IC (R = 0.580**); MC negatively correlates with TB (*R* = −0.333*) and C (*R* = −0.736**), while positively correlating with H (*R* = 0.390*); TB positively correlates with C (*R* = 0.558**) and TP (*R* = 0.650**), but negatively correlates with IC (*R* = −0.368*); and TP negatively correlates with IC (*R* = −0.358*). In the case of ethanol extract of dragon fruit peel powder (Figure 10), observed correlations include: MC positively correlating with TB (*R* = 0.530**) but negatively correlating with L (*R* = −0.764**), H (*R* = −0.499**), TP (*R* = −0.655**), IC (*R* = −0.460**), and ST (*R* = −0.397*); TB negatively correlates with L (*R* = −0.495**), H (*R* = −0.724**), TP (*R* = −0.700**), and ST (*R* = −0.522**), yet positively correlates with C (*R* = 0.394*); L positively correlates with H (*R* = 0.590**) and TP (*R* = 0.732**), but negatively correlates with C (*R* = −0.602**); H positively correlates with TP (*R* = 0.744**) and ST (*R* = 0.400*), while negatively correlating with C (*R* = −0.469**); C significantly negatively correlates with TP (*R* = −0.603**); TP significantly correlates with IC (*R* = 0.385*) and ST (*R* = 0.529**); IC positively correlates with ST (*R* = 0.918**). The correlation analysis sheds light on the interrelationships between these stability parameters, providing valuable insights into the behavior of the ethanol extract of dragon fruit peel and juice powders during storage.

## 4 CONCLUSION

The results highlight the distinct behavior: peel‐derived betacyanin exhibits a linear response to storage‐induced changes, whereas pulp‐derived betacyanin exhibits a relatively fluctuating trend. This disparity is attributed to the intricate composition of compounds within pulp‐based dragon fruit powder, contrasting with the peel. Notably, the brownish hue of peel‐derived dragon fruit powder results from prolonged production and heightened exposure to oxidizers. When maltodextrin is used as a coating material in peel and pulp dragon fruit powder manufacturing, drier powders with the potential for improved shelf stability are produced. Peel‐derived dragon fruit powder enriched with maltodextrin boasts elevated total phenolic content, underscoring its antioxidant potential. Furthermore, the combination of maltodextrin and gum arabic significantly elevates phytochemical levels of total phenol and betacyanin in pulp‐derived powders. These discoveries deepen our comprehension of betacyanin's stability and functional attributes through its sources. This discovery promises greater efficacy and applicability for the essential betacyanin components found in dragon fruit. Further research needs to be carried out to carry out a more specific comparative identification of the betacyanin component between dragon fruit pulp and juice and to find more apparent differences between the two types of ingredients.

## AUTHOR CONTRIBUTIONS

**Bambang Nurhadi:** Conceptualization (equal); data curation (equal); formal analysis (equal); funding acquisition (equal); methodology (equal); supervision (equal); writing – review and editing (equal). **Muhammad Abdillah Hasan Qonit:** Conceptualization (equal); data curation (equal); investigation (equal); methodology (equal); writing – original draft (equal); writing – review and editing (equal). **Syariful Mubarok:** Conceptualization (equal); data curation (equal); supervision (equal); writing – review and editing (equal). **Rudy Adi Saputra:** Data curation (equal); formal analysis (equal); methodology (equal); supervision (equal).

## FUNDING INFORMATION

This research was funded by the Ministry of Research and Technology/National Research and Innovation Agency.

## CONFLICT OF INTEREST STATEMENT

The authors declare no conflict of interest.

## Data Availability

Data is contained within the article.
